# Effects of Germination on the Nutritional Profile of Five Distinct Pea Varieties

**DOI:** 10.3390/molecules30153114

**Published:** 2025-07-25

**Authors:** Hojjat Abdollahikhamene, Shirin Kazemzadeh Pournaki, Clifford Hall

**Affiliations:** 1 Department of Dairy and Food Science, South Dakota State University, Brookings, SD 57007, USA; hojjat.abdollahikhamene@jacks.sdstate.edu; 2 Department of Food Science and Technology, University of California, Davis, CA 95616, USA; spournaki@ucdavis.edu

**Keywords:** pea, germination, B vitamins, protein, protein digestibility corrected amino acid score (PDCAAS)

## Abstract

The effects of germination on pea composition have been established. However, the effects of germination on the nutritional profile of different pea varieties have not been extensively reported. Therefore, five varieties (Passion, Greenwood, Durwood, Agassiz, and Treasure) of peas were germinated for up to six days, and their nutrient profiles, protein digestibility, in vitro protein digestibility corrected amino acid score (IV-PDCAAS), and antioxidant activity (DPPH) were determined. In addition, B vitamins were determined for the first time in most of the varieties evaluated. Germination enhanced protein digestibility across all varieties, whereas IV-PDCAAS tended to decrease with increasing germination day. The impact of germination on starch content varied, with decreasing percentages found in some varieties and increased percentages found in others. Soluble fiber increased and insoluble fiber decreased with increasing germination days. Thiamine and niacin tended to increase with increasing germination day, while pyridoxine and folate decreased. The radical scavenging activity of the germinated peas increased with increasing germination days. Overall, germination tended to improve the nutritional composition of peas, with only a few exceptions. Furthermore, the interaction effects between variety and germination day support the importance of knowing both variety and length of germination when creating germinated pea products.

## 1. Introduction

Pea (*Pisum sativum* L.) is one of the major legumes widely cultivated around the world, especially in Canada, China, northern Europe, Russia, and the United States. The Food and Agriculture Organization of the United Nations estimated that the world pea production was approximately 13.8 million metric tons in 2023 [1]. Peas are generally a rich source of dietary protein and carbohydrates, including starch and fiber, as well as vitamins, while being low in fat [2]. In addition, peas are a rich source of essential vitamins [3], including thiamine (B1), riboflavin (B2), folate (B9), pantothenic acid (B5), biotin (B7), pyridoxine (B6), along with fat-soluble vitamins such as phylloquinone (K1), β-carotene, and α-tocopherol [4]. However, their nutritional composition can vary depending on factors such as the type and variety of pea, growing environment, processing techniques, and the size of the flour particles [5,6]. For example, wrinkled green pea varieties had a higher total dietary fiber content (19.5%) compared to wrinkled yellow peas (17.5%) [7]. Ultrafine grinding of pea seeds increased their soluble dietary fiber (SDF) content from 1.3 to 5%, which supports the impact of processing on nutrient composition [8]. Another process that can impact nutrient content is germination. Over the last decade, germinated seeds have been recognized as a functional food, despite their consumption having existed for millennia [9].

Germination, also known as sprouting or malting, is a cost-effective strategy for addressing the nutritional limitations of grains and legumes by improving the digestibility and bioavailability of nutrients [10,11]. In the traditional plant growth cycle, germination ends seed dormancy and results in the activation of endogenous enzymes such as protease, amylase, and lipase [12]. Products such as amino acids, simple sugars, and fatty acids result from the enzymatic breakdown of complex molecules, such as proteins, carbohydrates, and lipids, respectively. The resulting products are generally easier to digest and are more bioavailable [13,14,15]. Furthermore, bioactive compounds such as polyphenols, gamma-aminobutyric acid, flavonoids, and tannins are more readily bioavailable in germinated cereals and pulses due to their release from being bound to the cell wall, for instance, which, in a free form, contributes to improved bioavailability and health-promoting activities [16,17,18]. Furthermore, many secondary compounds undergo biochemical modifications during the germination process, leading to improved bioavailability [19,20]. Germination has also been shown to impact water-soluble vitamins significantly [21,22]. Researchers have linked the bioactive compounds to a reduced risk of noncommunicable diseases, including cardiovascular disease, cancer, gastrointestinal disorders, diabetes, and obesity [23]. From a functional food perspective, the benefits of consuming germinated foods include improved management of metabolic disorders, such as diabetes, as well as broader health benefits [9].

During germination, the biochemical and structural transformations that occur are influenced by factors such as legume type, sprouting conditions, and enzymatic activity [17]. At the same time, antinutritional factors such as phytic acid, protease inhibitors, and oligosaccharides are reduced compared to raw, ungerminated grains, resulting in improved nutrient absorption and overall nutritional quality [24,25,26]. Germination also improves the physicochemical and sensory properties of grains [27]. The benefits of germination have been documented. However, many factors can influence the outcome of the germination process. One factor often overlooked, especially in pulses, is the impact of pulse variety on the germination process outcome. As a result, the study undertaken focused on using different pea varieties in the germination process. Furthermore, the duration of the germination, up to six days, was also a focus in establishing the appropriate germination time.

The objective of this study was to determine the impact of pea variety and duration of germination on the nutritional quality of different pea varieties, focusing on the proximate composition and associated parameters, such as protein digestibility, in vitro protein digestibility-corrected amino acid score (IV-PDCAAS), vitamin B content, and antioxidant activity. The primary hypothesis, based on previous research, is that a reduction in the percentage of protein, fat, ash, and starch will occur, while the concentrations of B vitamins, protein digestibility, IV-PDCAAS, and antioxidant activity will increase. An additional hypothesis is that each variety will undergo a similar pattern of changes and that the most significant changes will occur on day six of germination.

## 2. Results

### 2.1. Proximate Composition

#### 2.1.1. Effect of Variables on Composition

With few exceptions, significant effects of both variety and day on nutrients were determined (Table 1). Furthermore, interactions between variety and germination day were found for nutrient concentrations, except for protein and soluble fiber (Table 1). However, the protein content was significantly influenced by both variety and day (*p* < 0.05) separately. Soluble dietary fiber was only influenced by the length (day) of germination and was not influenced by variety or the interaction between variety and day (Table 1). Although the interaction effect was observed for fat and total starch, the main effect of day was not significant (*p* = 0.06), indicating that variety was the basis for the significant interaction effect.

**Figure 1:**
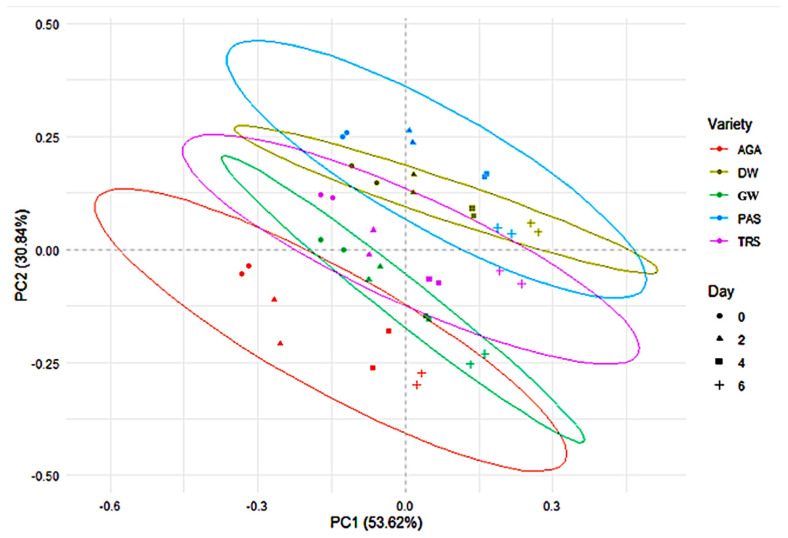
Principal component analysis (PCA) plot of non-germinated and germinated peas for five varieties over six days of germination based on compositional data.

The impact of variety and germination can be further visualized in the principal component analysis (PCA) plot (Figure 1). Collectively, 84% (i.e., 53.62% and 30.84% for PC1 and PC2, respectively) of the total variance could be explained by the first two PCs. However, as the germination day increased, PC1 became a more significant factor in causing the change in nutrients. The ellipses indicate the 95% confidence interval, showing that while varieties differ in their overall nutrient transformation patterns, some overlap is observed. The Agassiz (AGA) variety tended to overlap least with the other varieties and showed the most overlap with the greenwood (GW) variety as the number of germination days increased. Durwood (DW) and passion (PAS) varieties had patterns that were the most similar over the course of germination. Another interesting observation was that GW and treasure (TRS) initially overlapped, but as germination time increased, they became separate. These pattern changes indicate that the nutrient compositions of these two varieties were impacted differently as germination time increased. Overall, both variety-specific and time-dependent changes in nutritional composition occurred during the germination process. The broad changes observed were grounded in changes in nutrient composition (Table 2 and Table 3).

#### 2.1.2. Nutrient Composition

The moisture content in all pea varieties increased from an initial mean of 9.2% to a final percentage of 16.8% (Table 2). The increase was expected due to the addition of water and the humid germination conditions [28]. The PAS variety had the lowest moisture content at time 0, while AGA had the lowest on day 6. In both cases, the values were significantly lower than other samples (Table 2). The DW and TRS varieties had the highest (*p* ≤ 0.05) moisture contents by day 6. Overall, no single variety consistently had lower or higher moisture content over the course of the study.

Ash content decreased significantly (*p* ≤ 0.05) during germination in all varieties (Table 3). In contrast to moisture content, the AGA variety had significantly higher ash contents at each of the time points compared to other varieties. The other four pea varieties had initial and final ash contents that were significantly different; however, none of the four remaining varieties had an ash content that was clearly different at every time point, like in AGA. Protein percentages in all varieties increased over the course of the germination study. However, the increase from day 0 to day 6 was only significant (*p* ≤ 0.05) for the AGA and DW varieties. The PAS and DW varieties had consistently higher protein percentages, although the values were not always statistically significant. The AGA variety had the lowest protein percentage during the germination period, although only the protein percentages at day 2 were significantly different from those of all other varieties.

Fat content fluctuated among varieties (Table 3), with AGA, GW, and PAS increasing during the germination period, while DW and TRS decreased. However, the changes from day 0 to day 6 were not significant within each respective variety. The GW variety maintained the highest fat percentage among the varieties, although only the sample from day 6 was significantly different from the other varieties, except AGA. The same pattern was observed for total starch, where starch levels increased in AGA, GW, and PAS varieties and decreased in DW and TRS. Like fat content, the change in starch percentage was not significant within each respective variety. Only the AGA day six sample had a significantly higher starch percentage compared to the other samples, except GW. In general, AGA tended to have the highest starch percentage among varieties at each time period, although in a few cases the value was not significant. As indicated, DW and PAS varieties decrease with increasing germination time. The DW underwent an approximate 2 percentage point reduction in starch while PAS underwent an 11 percentage point reduction in starch content.

**Figure 2:**
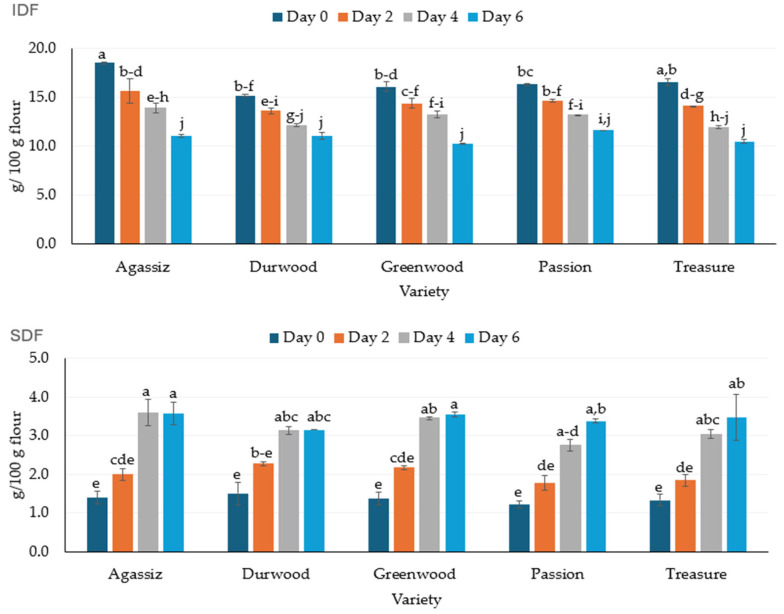
The changes in insoluble dietary fiber (IDF) and soluble dietary fiber (SDF) over a six-day germination of five pea varieties. The line bars represent standard error, while the letters above each bar reflect significant differences at *p* ≤ 0.05 between samples within fiber type.

The total dietary fiber (TDF) content decreased significantly with increasing germination time for all varieties (Table 3). The decrease was reflected in a significant (*p* ≤ 0.05) reduction in the percentage of insoluble dietary fiber (IDF) across pea varieties (Figure 2). The reduction in IDF ranged from 26% to 39% in the DW and AGA varieties. However, the IDF percentage was not significantly different among varieties at day 6. In contrast to IDF, the percentages of soluble dietary fiber (SDF) across various pea varieties increased significantly over germination days up to day four (Figure 2). The SDF was not significantly different at days four and six, regardless of the variety. The SDF of the PAS variety increased from 1.2% on day 0 to 3.8% on day 6, a 217% increase in SDF. The SDF content of other varieties also increased between 107% and 179%. When considering both the changes in IDF and SDF, the overall reduction in total dietary fiber was between 14 and 27%. The change in fiber can also be illustrated by the change in the IDF/TDF ratio. In all varieties, the ratio decreased with increasing germination time. The IDF/TDF ratio decreased from 92.2% to 74.2% in GW, 94% to 75.5% in AGA, 92.6% to 76.9% in TRS, 91% to 77.8% in DW, and 93.1% to 77.5% in PAs between day 0 and day 6.

### 2.2. B Vitamin Concentrations

**Figure 3:**
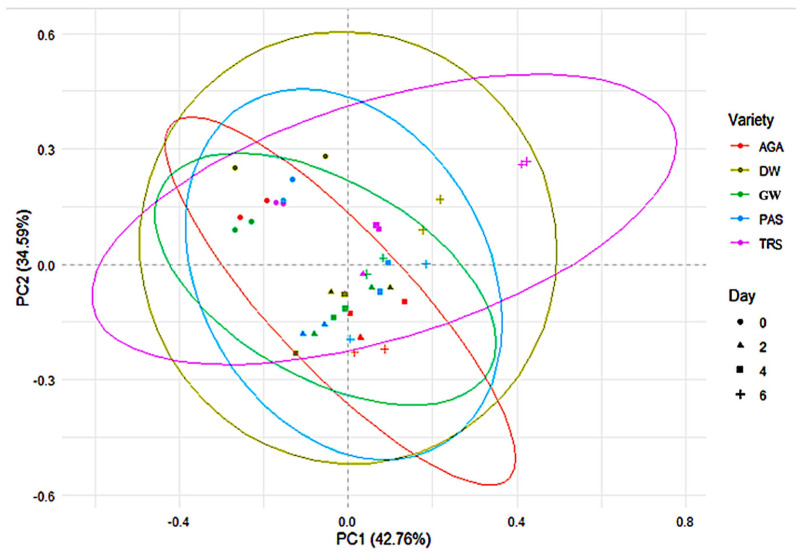
Principal component analysis (PCA) plot of non-germinated and germinated peas for five varieties and six germination days based on vitamin B data.

Vitamin concentrations (thiamine, riboflavin, niacin, pyridoxine, and folic acid) were significantly affected by day (*p* < 0.05) and the variety × day interaction (*p* < 0.05) during germination. Differences in composition were noted for varieties; the variety effect contributed less to the differences compared with the day and interaction effects. This indicates that these vitamins are primarily influenced by germination duration rather than variety (Figure 3).

The impact of variety and germination can be further visualized in the PCA plot (Figure 3). The PCA plot shows that 42.76% (PC1) and 34.59% (PC2) of the total variance could be explained by the first two PCs. The ellipses indicate the 95% confidence interval and show that the clusters for different varieties were not compact as in the nutrient PCA plot (Figure 1). This indicates that variety is a less significant factor in differentiating B vitamin composition. Day-based clustering revealed that the non-germinated peas clustered separately from the germinated samples. Furthermore, later stages (Day 6) of germination were less homogeneous, as illustrated by the wider distribution in the PC plot. Overall, time-dependent changes in vitamin composition occurred during the germination process. However, the broad changes observed in the PCA plot were primarily driven by changes in vitamin composition over time, and to a lesser extent, by variety (Table 4).

The impact of variety and time on vitamin B concentration was dependent on the vitamin B analogue being evaluated. Folic acid and pyridoxine concentrations generally decreased with increasing germination days. In contrast, niacin, riboflavin, and thiamine concentrations increased (Table 4). The folic acid content varied substantially and decreased significantly over the 6-day germination period regardless of the variety. However, the percentage reduction did vary based on the variety. The AGA variety lost 93% of the folic acid, while TRS, DW, PAS, and GW lost 41%, 74%, 78%, and 85%, respectively. Pyridoxine concentration was the highest among the B vitamins. Like folic acid, the percentage reduction over the germination period varied based on variety. After 6 days, the AGA variety lost 91% of the pyridoxine compared to the non-germinated pea. The loss in pyridoxine reached 67%, 67%, 76%, and 87% for DW, GW, TRS, and PAS, respectively. All these losses were significant between day 0 and day 6. However, DW had significantly higher pyridoxine than AGA and PAS on day six.

Niacin content increased across all varieties during germination, with TRS having the highest concentration (1434 µg/100 g) on day 6. This represents a 101% increase compared to the day 0 sample and was significantly higher than other varieties at day 6. Increases of 13%, 36%, 43%, and 48% were observed for the AGA, DW, GW, and PAS varieties, respectively. Thiamine concentration generally increased over the germination period, particularly in the TRS variety, which peaked at 432.1 µg/100 g by day 6 (Table 4). This was an increase of 79%. The increase in thiamine between day 0 and day 6 was only significant (*p* ≤ 0.05) for the TRS variety. Increased thiamine concentrations of 24, 26, and 30% were observed for the AGA, DW, and GW varieties, respectively. Only the PAS variety had lower (11%) thiamine content at day 6. However, the PAS sample from day 4 had 5% more thiamine compared to the time 0 PAS sample. Regardless, the thiamine concentration in PAS was not significant over the course of germination.

Riboflavin concentrations varied significantly among varieties and days. In all varieties, a significant decrease in riboflavin was observed on day 2 of germination, followed by a non-significant increase in riboflavin concentrations on day 4 and in some varieties, on day 6 (Table 4). Riboflavin concentrations were significantly higher in the day 6 germinated samples of TRS, GW, and DW (84, 83, and 78%, respectively) compared to their respective non-germinated samples. A 6% and 65% reduction in riboflavin was observed for the PAS and AGA varieties. However, only the reduction in riboflavin in the AGA was significant compared to the non-germinated sample.

### 2.3. DPPH Radical Scavenging

**Figure 4:**
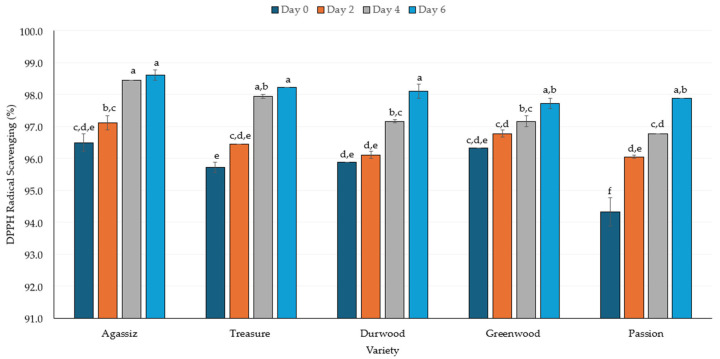
The antioxidant activity (i.e., DPPH radical scavenging) of five pea varieties germinated over 6 days. The line bars represent standard errors, while the letters above each bar reflect significant differences at *p* ≤ 0.05 between samples.

Significant effects of variety (*p* < 0.5), germination day (*p* < 0.5), and the interaction (*p* < 0.05) were determined for the antioxidant activity (i.e., DPPH radical scavenging). This indicates that the variety and duration of the germination period had an impact on antioxidant activity. Regardless of the variety, the peas germinated for 6 days had significantly higher DPPH radical scavenging activity than samples from day 2 or the non-germinated samples (day 0). Furthermore, in DW and PAS varieties, the 6-day germinated samples had significantly higher scavenging activity than the 4-day germinated samples. The non-germinated samples generally had the lowest DPPH radical scavenging activity, although in some cases the value was not statistically different from the samples from the 2-day germination (Figure 4).

In addition to germination duration, differences in DPPH radical scavenging activities were observed among varieties. The AGA variety had DPPH radical scavenging activity that was slightly higher than that of other varieties, although in most cases the values were not significantly different. When compared to their non-germinated samples, the percent increase in radical scavenging activity was 1%, 2%, 2%, 3%, and 4% for GW, AGA, DW, TRS, and PAS, respectively.

### 2.4. Protein Digestibility and In Vitro Protein-Corrected Amino Acid Score (IV-PDCAAS)

**Figure 5:**
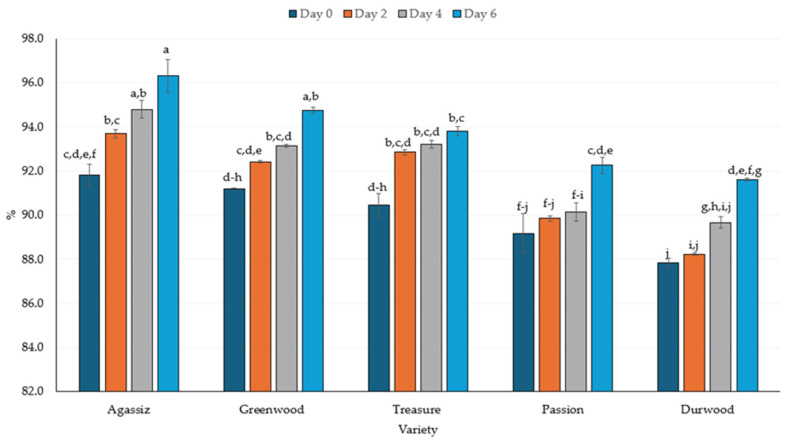
The in vitro protein digestibility (%) of five pea varieties germinated over a 6-day period. The line bars represent standard error, while the letters above each bar reflect significant differences at *p* ≤ 0.05 between samples.

Protein digestibility and IV-PDCAAS were significantly influenced by both factors, though the interaction effect was not significant for digestibility (Table 5). However, the interaction between variety and germination duration was significant for PDCAAS. Protein digestibility increased significantly during the 6-day germination period (Figure 5). The AGA and DW varieties had the highest and lowest protein digestibility values, at 96.3% and 91.6%, respectively, on Day 6. These values represent an increase of 5% and 4%, respectively, compared to their respective non-germinated samples. The GW, TRS, and PAS varieties, after 6 days of germination, also had higher protein digestibility values (i.e., 4%, 4%, and 3%, respectively) compared to their respective non-germinated forms. Overall, significant differences in protein digestibility were observed among varieties.

**Figure 6:**
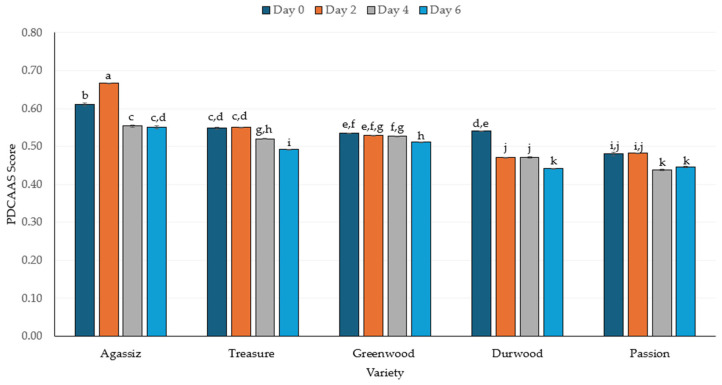
The protein-corrected amino acid score (PDCAAS) of five pea varieties germinated over 6 days. The line bars represent standard errors, while the letters above each bar reflect significant differences at *p* ≤ 0.05 between samples.

The IV-PDCAAS had a downward-trending pattern with increasing duration of germination (Figure 6). The AGA variety initially had the highest IV-PDCAAS (0.60), which increased to 0.67 on day 2, but decreased slightly over the remaining days. The IV-PDCAAS scores were the same on day 0 and day 2 for the TRS and GW varieties; however, the IV-PDCAAS scores decreased gradually through days 4 and 6 of germination. In contrast, PAS and DW consistently had significantly lower IV-PDCAAS scores, particularly DW, which dropped to 0.44 by day 6, representing a 19% reduction in the IV-PDCAAS score. Reductions of 4%, 8%, 9%, and 10% in IV-PDCAAS were observed in the GW, PAS, TRS, and AGA varieties, respectively. The germinated AGA and GW varieties, after 6 days of germination, had higher protein digestibility values compared to their respective non-germinated forms. This higher protein digestibility likely contributed to the higher IV-PDCAAS scores.

## 3. Discussion

### 3.1. General Composition

As expected, the moisture content of the peas increased over the germination duration, with the maximum moisture observed in the six-day germinated peas (Table 2). Moisture percentage increased sharply for lentils between days 2 and 4 of germination, whereas for the yellow pea, the increased moisture percentage was noted between days 4 and 6 [17]. There was a gradual increase in the moisture content of chickpea obtained from 6-day germination of whole chickpeas [17].

The ash contents (2.2–3.0%) of the peas used in the current study are within the range of ash contents previously reported for peas [29,30,31,32,33]. In addition, the variability in ash content of the five varieties tested in this study agrees with significant differences in ash concentration among various dry pea cultivars reported previously [29,34]. Due to these differences, using multiple varieties of a commodity allows for a better representation of outcomes that could occur when subjecting the commodity to processing.

Ash content significantly decreased after two days of germination, reaching its lowest values at six days for all varieties (Table 2). Initial ash content ranged from 2.2% to 3% at day zero and decreased to 1.9% to 2.5% after six days of germination. A consistent trend of decreasing ash content was observed across all varieties. This observation agrees with a slight fluctuation in ash content of germinated peas [17], cowpeas, and chickpeas [35]. The loss of ash may be due to the leaching of minerals during soaking and exposure to moisture during germination [35]. Furthermore, phytate phosphorus can be converted to the more water-soluble phosphorus form during germination [36], contributing to a loss by leaching into the water used to maintain hydration of the germinating pea. In contrast, pigeon pea (*Cajanus cajan*) had an increased ash content after a 72 h germination [22]. These authors found increases in calcium, iron, and zinc. One reason for the increased minerals and ash content could be associated with the water used during the germination process, where potable sources have varying calcium and iron contents. In the current study, distilled water was used; thus, no additional minerals were added due to the water source while maintaining a humid and hydrated condition during germination.

The protein contents (22.9–27.8%) of the peas used in the current study are within the range of protein contents previously reported for peas [37,38,39]. Varietal differences in protein content of non-germinated samples in this study are supported by data from the literature [38,40]. In the current study, PAS and DW had higher protein contents among the five varieties tested. These two varieties also had higher protein contents over the course of the germination process, which supports the significant varietal effect (Table 1).

Overall, the percentage protein of the germinated pea increased from 8% (PAS) to 21% (DW). The observed increase in protein has been observed in germinated peas [17,41] and other pulses, such as chickpea, faba bean, horse gram, lentil, and lupin [17,41,42,43,44,45]. In addition, the results of the current study are supported by previous studies, where increased protein percentage was pea variety-dependent. For example, 21.2, 22.9, and 25.5% increases in protein content were observed for the pea varieties ramrod, agra, and cero, respectively [46,47]. In the current study, distilled water was used during the germination process. The water source is important, since protein production requires a nitrogen source. Thus, protein synthesis is not likely the reason for the increased protein during germination. Instead, the reduction of other seed components and a concomitant increase in protein percentage relative to dry weight was the primary reason for the increased protein content during germination [17].

A majority of authors have reported increased protein concentrations in legume sprouts during germination, while others observed no changes or reduced levels [48]. During germination, proteases break down storage proteins into free amino acids and small peptides, which the developing radicle then uses to synthesize functional and structural proteins needed for seedling growth [49]. Thus, a loss of protein would be expected if much of the free amino acid or small peptides are not taken up by the growing seedling. In the current study, an increased percentage was observed; thus, it was most likely that protein content was based on a reduction of other components and that the seedlings had not sufficiently progressed in their life cycle to cause a degradation of the protein via enzymatic processes.

All non-germinated pea varieties had fat contents that fell between 1.1 and 2.4% (Table 3). The fat content of the varieties used in the current study agrees with the 1–4% lipid content typically reported for peas [37,38,50]. The variety effect (Table 1) was found among the five varieties tested. The GW variety consistently maintained the highest fat percentage (2.4 to 2.6%) over the duration of germination. The AGA followed a similar small, but non-significant, increase in fat content over the six-day germination. Furthermore, the day of germination did not significantly affect fat content in other varieties. A small, but non-significant, reduction in fat was observed in the TRS, DW, and PAS varieties. The expectation was that the fat would decrease during germination, as reported previously [17]. A decrease was expected due to the enzymatic hydrolysis of the triacylglycerol, which releases free fatty acids that can be utilized as an energy source by the mitochondria and cytosol during early germination [51,52]. Thus, the minimal changes that occurred may be related to increases or decreases in other components during the germination process.

The starch contents (45.7–59.5%) of the peas used in the current study (Table 2) tended to be higher than the range in starch contents (33–47%) previously reported for peas [17,29,53,54] but similar to those reported by Mohammed et al. [40]. Starch contents varied among varieties and during germination (Table 2). The starch content of the AGA variety reached its peak of 59.5% on day six. The PAS variety had the most significant reduction in starch content, to 40.1%, by day six. The DW, PAS, and TRS varieties at day 6 of germination followed the expected outcome of a reduction in starch percentage.

Starch breakdown, caused by increased α-amylase activity during germination, has been widely documented, along with a reduction in starch content in germinated peas [17,55,56]. Oligosaccharides and glucose are just a few examples of breakdown products that are likely to occur, resulting in the reduction of total starch [57]. The breakdown of starch during germination is initiated by phosphorylase, which yields glucose-1-phosphate that is used for energy and cell wall growth [15,58]. α-Amylase activity in cow pea and lentil increased to 666% and 570%, respectively, after 72 h of germination compared to raw seeds [56]. The degree of starch digestibility by α-amylase in pulses is often limited to only 25 to 35% due to structural characteristics of the starch granules [59]. Thus, complete starch digestion may not occur during the limited germination time used in the current study. Over a 6-day germination period, there was a decrease in the starch content in peas by 2.87 g/100 g or 7% [60]. In the current study, starch reductions of 2, 5, and 21% were observed for the TRS, DW, and PAS varieties, respectively. In general, the percentage of reductions is consistent with other studies on peas [21,56].

The total dietary fiber (TDF) for the five varieties tested ranged from 16.6 to 19.9% in the DW and AGA varieties, respectively (Table 3). The data for non-germinated peas in the current study are in agreement with previously reported data [61,62,63,64,65]. The IDF percentages ranged from 15.1 to 18.5% in the non-germinated samples, corresponding to the DW and AGA varieties, respectively (Figure 2). For SDF, the PAS and DW varieties had the lowest and highest values, respectively, on day 0. Overall, the IDF and SDF are slightly higher than or in agreement with previously reported fiber data [61,62]. Furthermore, the previously reported data supports the influence of variety on fiber (i.e., TDF, IDS, SDF) content observed in the current study.

For all varieties, the general trend was a reduction in the TDF percentage (Table 3). The reduction in TDF ranged from 14% in DW and PAS varieties to 27% in the AGA variety. In contrast, other researchers have reported increased fiber content in germinated pulses [42,44,66]. In the current study, reductions in TDF were associated with the reduction in IDF, given that SDF increased over the six-day germination period (Figure 2). A 34% reduction in IDF was observed across all varieties, with DW and PAS having the highest (41%) and lowest (22%) reductions, respectively, among the varieties. Thus, it is not clear why there is a divergent outcome between previous reports and the current study.

The SDF increased on average by 161%, with the SDF content of PAS and DW increasing by 107% and 179%, respectively, over the six-day germination period. The SDF results are in agreement with data previously reported on pea SDF [67]. Overall, the significant reduction in IDF indicates that the germination process may be breaking down some of the insoluble fibers, potentially converting them into soluble fibers or smaller fragments that do not contribute to the overall dietary fiber content [22].

### 3.2. B Vitamins

The B vitamins have not been studied extensively relative to pulse variety and pulse germination. The concentrations of folate, niacin, pyridoxine, riboflavin, and thiamine were 352–519, 456–714, 1151–1467, 77–156, and 207–279 µg/100 g, respectively. The vitamin B concentrations found in the raw pea in the current study partially agree with data previously reported [21,68,69,70,71]. For example, a similar riboflavin concentration (150 µg/100 g) but a different thiamine concentration (730 µg/100 g) was reported by Urbano et al. [21] compared to results from the current study. The thiamine concentration of 340 µg/100 g [15] was in closer proximity to concentrations found in the current study. The folate content reported in field peas was 26–202 μg/100 g [66,67,68,69], which is significantly lower than the folate determined in the current study. Sen Gupta et al. [70] reported differences among pea market classes, in which yellow pea (41–55 μg/100 g) had lower folate levels than green pea (50–202 μg/100 g). These data, along with the data reported in the current study (Table 4), support a varietal effect, suggesting that differences in reported vitamin B concentrations may be dependent on the variety evaluated.

The germination process produced divergent outcomes depending on the vitamin analyzed (Table 4). Folate and pyridoxine concentrations decreased with the increase in time of germination. In contrast, minimal changes or increasing concentrations of niacin and thiamine occurred with increasing germination duration. Riboflavin resulted in both increasing and decreasing concentrations depending on the variety. Overall, the results of the current study follow similar trends to those in the literature regarding changes in vitamin B concentrations.

Pyridoxine concentration decreased significantly over the course of germination, and by day 6, the pyridoxine concentration dropped anywhere from 67% to 91% compared to the non-germinated pea. This may reflect the instability of pyridoxine or possible leaching into the water. However, the decrease in pyridoxine during germination was also observed in sorghum [72]. In contrast, pyridoxine increased by 51% over the course of a 5-day germination of lentils [73].

Folate content varied significantly and decreased substantially over the six-day germination (Table 4). All varieties followed the same pattern of folate reduction. The total folate content in mung bean seeds increased during the first four days of germination by approximately 400 µg/100 g and then decreased on day 10 of germination [74]. Increasing folate concentrations were observed in cowpeas and lentils over a four- and five-day germination process [73,75]. Additionally, folate concentrations increased in peas, lentils, and beans [76]. Increased folate contents in lupin germinated for nine days further support the beneficial impact of germination on folate content [77]. As a result, one possible explanation for the reduction in folate might be that, in the current study, multiple forms of folate were not determined, as was performed in other studies.

The niacin content increased for all varieties, with TRS having the highest concentration (1545 µg/100 g) on day 6. Significant increase in niacin was also observed in lentils after a 5-day germination [73]. Significant increases in niacin concentration were observed during the germination of Tartary buckwheat [78]. Tarr and Arditti [79] reported that niacin biosynthesis in corn seedlings occurred via oxidative degradation of tryptophan. Thus, the increased niacin concentration in germinated peas might be due to this biosynthetic process. The results observed in the current study are supported by increased niacin in other germinated commodities.

A possible reason for the increased niacin could be the breakdown of complex forms into more bioavailable ones that were produced during germination [18]. Ghavidel and Prakash [35] reported that the thiamine content of cowpea and pea increased from 640 µg/100 g to 850 µg/100 g and 340 µg/100 g to 510 µg/100 g after 24 h of germination, respectively. Thiamine content in lentils increased approximately 150% (154 µg/100 g to 334 µg/100 g) after 24 h of germination [68]. In contrast, thiamine increased during the first two days of lentil germination, then reduced through day 5 [73]. This same trend was observed in two varieties (AGA and TRS) in the current study, while the other three varieties had higher concentrations with increasing days of germination.

Riboflavin concentration varied significantly among varieties and days. In all varieties, a significant decrease in riboflavin was observed on day 2 of germination, followed by increasing riboflavin concentrations until day 6 in all varieties except AGA (Table 4). The observed increase in riboflavin is supported by published research. Hsu et al. [41] reported that riboflavin increased by 108, 22, and 40% in yellow peas, lentils, and faba beans, respectively, after 4 days of germination. A 134% increase in riboflavin was also reported in lentils [73].

### 3.3. Antioxidant Activity

Antioxidant activity, measured by DPPH scavenging ability, remained consistently high across all varieties, with AGA having the highest DPPH radical scavenging by day 6 (98.6%) (Figure 4). The percentage increase in radical scavenging activity was 1%, 2%, 2%, 3%, and 4% for GW, AGA, DW, TRS, and PAS, respectively, compared to the non-germinated samples. The increased DPPH radical scavenging is in agreement with other reported increases in antioxidant activity of germinated peas [80,81,82], lentils [81], pigeon peas [83,84], and moth beans [85], among others. In contrast, decreasing antioxidant activity was reported for lentil, black gram, mung, and pigeon pea [80,85]. The contrasting outcomes of antioxidant activity are likely correlated with the composition of the phenolic compounds.

The germinated pulses with higher DPPH radical scavenging compared to non-germinated pulses also had higher total phenolic compound values [81,83,84,85]. The length of germination of peas up to seven days was reported to result in the highest antioxidant activity as well as total phenolic and flavonoid contents [82]. However, extending the germination period to 10 days resulted in decreased antioxidant activity and phenolic compound levels in germinated peas. Germination also promotes the release of bound phenolic compounds, resulting in the formation of new free and soluble phenolics that can more effectively impart antioxidant activity [80,82,86,87]. In the current study, the resulting increase in DPPH radical scavenging of the germinated peas was expected (Figure 4). However, the influence of variety was less important since the day six germinated peas had, statistically, the same radical scavenging activity among varieties.

### 3.4. Protein Digestibility and PDCAAS

In the current study, the increase in IVPD was approximately 3–5% for the germinated peas compared to non-germinated peas (Figure 5). The increase in digestibility agrees with earlier research, which indicated that germination improved protein digestibility [88,89]. The 3–5% increase in protein digestibility found in the current study agrees closely with the 2% increase in IVPD reported by Setia et al. [88]. The short-term germination of yellow peas and faba beans resulted in higher protein digestibility [88,90]. In contrast, the IVPD of yellow pea did not significantly increase after a three-day [64] or four-day germination [91]. However, germination followed by cooking improved IVPD [92]. For chickpeas, IVPD increased from 68% in raw seeds to 70% after soaking and further improved to 72, 76, and 79% after 3, 4, and 5 days of germination, respectively [66]. The IVPD of pigeon pea increased by 19 and 41% after germination at 25 and 35 °C, respectively [84]. Chinma et al. [22] also reported a 14% increase in IVPD of germinated pigeon peas. Although reports exist regarding the lack of improvement in IVPD, the majority of the studies indicate improved IVPD in various legumes.

Germination significantly improved the protein digestibility of chickpeas, as assessed by an in vitro digestion model (oral, stomach, duodenal, and brush border) [93]. These authors found increases in free alpha-amino nitrogen levels and oligopeptides, while the large, digestion-resistant peptides decreased in the germinated chickpea [93]. Other researchers support the breakdown of high-molecular-weight proteins into smaller, more digestible substances, thereby improving overall protein digestibility [89,94,95]. In addition to hydrolysis, germination can cause changes in the protein structure itself and reduce anti-nutritional factors such as protease inhibitors [89,96]. Weakening of protein–starch interactions has also been proposed as a possible mechanism of improved protein digestibility [97].

The general trend of decreasing IV-PDCAAS scores over the course of germination was observed (Figure 6). The reduction in IV-PDCAAS was lowest (4%) in the GW variety, while DW variety had the most significant reduction (19%) in IV-PDCAAS. A 14% reduction in IV-PDCAAS was reported for yellow pea, while no change in the IV-PDCAAS was observed in faba bean [88]. Germination alters the ratio of essential to non-essential amino acids, potentially increasing the availability of essential amino acids [63]. However, the lower IV-PDCAAS scores may be the result of a decline in limiting essential amino acids, despite an increase in IV-PD [98]. In contrast, germination of yellow pea resulted in a higher IV-PDCAAS, likely due to increased availability of the limiting amino acid [86]. However, this author did observe a decrease in IV-PDCAAS in germinated lentils. Overall, this indicates that germination did not increase protein nutritional quality [88].

Although all IV-PDCAAS scores declined over germination, the AGA variety had the highest IV-PDCAAS score. This variety also had the highest protein digestibility. Thus, this variety may be better suited for germination than other varieties tested since more of the protein quality was retained during germination.

## 4. Materials and Methods

### 4.1. Pea Germination

Two replicates of the pea varieties passion (PAS), greenwood (GW), durwood (DW), agassiz (AGA), and treasure (TRS) were obtained from the 2022 harvest year, cleaned, sorted, and stored at 4 °C in darkness until needed. The cotyledon colors of the pea were green for PAS and GW and yellow for DW, AGA, and TRS. The PAS and GW varieties were obtained from producers in the States of Idaho (replicate 1) and Washington (replicate 2). The AGA and DW varieties were obtained from producers in the States of North Dakota (replicate 1) and Montana (replicate 2). The TRS variety was obtained from producers in the States of Idaho (replicate 1) and Montana (replicate 2).

Prior to germination, peas in 500 g batches were soaked in 0.07% sodium hypochlorite for 30 min, followed by soaking in distilled water for 5.5 h to remove residual hypochlorite solution. This process was performed to facilitate surface sterilization of the peas. The surface-sterilized peas were placed on germination trays and germinated at 22–25 °C and 70–80% relative humidity in a dark environment. Samples (500 g) were collected on days 2, 4, and 6, ground with a food processor into a paste, and freeze-dried (Harvest Right, Salt Lake City, UT, USA). Samples (200 g) that were surface sterilized, soaked to remove hypochlorite residue, and dried but not germinated served as the control or day 0 sample. After drying, 200 g of pea seeds per variety were milled through a 0.5 mm screen using a UDY Cyclone Sample Mill (Direct Drive 3010–014, UDY Corp., Fort Collin, CO, USA). The samples were stored at −4 °C until analyses could be completed.

### 4.2. Composition Analysis

The moisture, protein, total starch, and ash contents were determined following the AACC approved methods 44–17.01, 46–30.01, 76–13.01, and 08–01.01, respectively [99,100,101,102]. Fat was determined by the Official Methods and Recommended Practices of the AOCS Official Method Ba 3-38 [103]. Fiber analysis was completed following the AOAC 991.43 [104] fiber analysis method on an Ankom automated TDF Dietary Fiber Analyzer (Ankom, Macedon, NY, USA).

### 4.3. In Vitro Protein Digestibility Corrected Amino Acid Score (IV-PDCAAS)

A Megazyme K-PDCAAS protein digestibility kit (Neogen Inc., Lansing, MI, USA) was used to determine protein digestibility and PDCAAS score [105]. Milled samples (500 mg) were digested using a series of gastric and intestinal enzymes provided in the K-PDCAAS protein digestibility kit. Following digestion, trichloroacetic acid (TCA) was added to facilitate the precipitation of peptides. The final steps in the Megazyme kit involved analysis of the peptides using L-glycine calibration standards and a colorimetric determination of amines at 570 nm (Equation (1)).

in vitro digestibility = (M × X + B)/100(1)

where the slope of the regression line is derived from literature-based reference values (1.1135). The X is the corrected primary amine concentration (CN) for each sample, and B is the Y-intercept of the regression equation, which adjusts for baseline differences in primary amine concentrations (74.125). The values of M and B are typically obtained from experimental calibration curves or literature references. The IV-PDCAAS was calculated using Equation (2).

Amino Acid Score = Protein Digestibility × Amino Acid Score(2)

### 4.4. Determination of B Vitamins by HPLC 

Thiamine hydrochloride, riboflavin tetrabutyrate, niacin, pyridoxine hydrochloride, and folic acid were sourced from TCI Chemicals (Portland, OR, USA), each with a purity exceeding 98%. β-Mercaptoethanol was obtained from Sigma-Aldrich (St. Louis, MO, USA), while HPLC grade acetonitrile and water were obtained from Fisher Scientific (Waltham, MA, USA). The mobile phases were filtered through PTFE membrane filters (0.45 µm pore and 25 mm diameter) prior to use. When necessary, HPLC grade water or mobile phase was used to dilute enzyme and B vitamin standards.

The extraction of B vitamins from pea samples was completed using a quad-enzyme extraction method [106]. Briefly, 50 mg of milled pea sample and 1 mL sodium phosphate buffer (pH 5.5) containing β-mercaptoethanol (2%) were mixed under subdued light to prevent photooxidation. This was followed by a series of heating and cooling steps, enzyme incubation steps, and centrifugation at 17,000× *g*. Full details of the method can be found in [106]. All extracted samples were filtered using 0.45 µm PTFE, pre-slit septa Captiva filter vials (Agilent, Santa Clara, CA, USA) before HPLC analysis.

Precaution to minimize light exposure during the extraction and standard vitamin preparation was taken to avoid B vitamin degradation. A 1260 Infinity Agilent HPLC system was used to separate the B vitamins on a Poroshell HILIC-OH5 column (Agilent, Santa Clara, CA, USA). The mobile phase included HPLC-grade water with 100 mM ammonium acetate, 0.5% acetic acid (A), and acetonitrile (B). The gradient began at 87% B for 0.5 min, decreased to 50% B over 6 min, and re-equilibrated to 87% B for 3 min. Detection at 260 nm and an Agilent CQL data analyzer (Agilent, Santa Clara, CA, USA) were used to quantify the B vitamins.

### 4.5. Antioxidant Activity 

The 2,2-Diphenyl-1-picrylhydrazyl (DPPH) radical scavenging assay, as described by Bhattarai and Janaswamy [107], was used to assess antioxidant activity. Flour (0.5 g) and absolute ethanol (10 mL) were shaken on a mechanical shaker at 100 rpm for 3 h. After centrifugation (2500× *g*), the supernatant (1 mL) was mixed with 0.1 mM DPPH solution (3 mL) and incubated in the dark for 30 min. Absorbance measurements were taken with a UV/Vis spectrophotometer (GENESYS 50, Thermo Scientific™, Waltham, MA, USA) at 517 nm. A solution of 3 mL of DPPH and 1 mL of ethanol served as a control. All solutions subjected to radical scavenging activity were calculated (Equation (3)) against a DPPH standard absorbance of 0.90.

%scavenging = (1 − (Absorbance of sample)/(Absorbance of control)) × 100(3)

### 4.6. Experimental and Statistical Analysis

Germination experiments were conducted in duplicate over six days, with samples collected at four intervals (0, 2, 4, and 6 days). Each batch of germinated pea flour was analyzed in duplicate (n = 4). Statistical analysis was performed using two-way ANOVA in RStudio 4.5.1, with Tukey’s least square differences (LSD) applied to establish mean separation. Interaction effects between germination day and pea varieties were evaluated and significance identified at *p* > 0.05. Principal Component Analysis (PCA) was used to identify patterns in the data. PCA helped simplify the dataset by reducing the number of variables, making it easier to identify the key factors that caused variation. The analysis was performed using the prcomp function in RStudio, and the results were represented using biplots.

## 5. Conclusions

A reduction in percentage protein, fat, ash, and starch was hypothesized. This hypothesis was supported by the reduction of ash and fiber contents during germination. Mixed results were observed for fat and starch contents, where some of the pea varieties had increased fat or starch contents while the other varieties had decreased nutrient contents. In the case of protein, the opposite outcome occurred as the percentage increased with longer germination times. The hypothesis that the concentrations of B vitamins, protein digestibility, IV-PDCAAS, and antioxidant activity would increase was partially supported. Both protein digestibility (IVPD) and antioxidant activity increased with increasing germination time for all varieties. Mixed outcomes were observed with B vitamins, where folate and pyridoxine decreased over the course of germination, whereas niacin increased as expected. Mixed results were observed for riboflavin and thiamine, where some varieties had increased concentrations of these vitamins while other varieties had decreasing concentrations over the course of germination. The reduction in IV-PDCAAS after germination was the opposite of the original hypothesis.

An additional hypothesis was that each variety would undergo a similar pattern of changes and that the most significant changes would occur on day six of germination. The most significant changes generally occurred on day six of germination, which supports the hypothesis. However, the varieties did not always undergo the same pattern of changes as described above. Thus, the observed outcomes did not support the hypothesis. With a few exceptions, the variety and day factors impacted the outcome of the experiment. This indicates that varieties and duration of germination were important factors in affecting the change in nutrient composition and associated parameters such as IV-PDCAAS. However, the environment where the peas were grown may have impacted the outcome. While the State, in the United States, where the peas were grown, was identified, a systematic approach to obtaining samples from the same locations was not performed. Therefore, expanding the research to assess environmental impacts on germination outcomes is encouraged and would further expand our knowledge of factors impacting germination.

Overall, with a few exceptions, the variety and day factors impacted the outcome of the experiment. This suggests that the variety and duration of germination were significant factors in influencing the change in nutrient composition and associated parameters, such as IV-PDCAAS. While some of the changes in nutrient composition were hypothesized correctly, others, such as increased B vitamins and IV-PDCAAS, were not. This study is the first to include multiple pea varieties, adding significance to germination research. Among the varieties tested, the AGA and TRS varieties tended to produce the most favorable results (e.g., higher protein digestibility and antioxidant activity) and might be varieties targeted for additional research.

## Figures and Tables

**Table 1:** Effects of the variety, day, and interaction between variables on the proximate composition of non-germinated and germinated peas.

Measurement	*p* Value		
	Variety	Day	Variety × Day
Moisture (%)	<0.05	<0.05	<0.05
Protein (%)	<0.05	<0.05	0.29
Fat (%)	<0.05	0.06	<0.05
Total starch (%)	<0.05	0.85	<0.05
Insoluble dietary fiber (%)	<0.05	<0.05	<0.05
Soluble dietary fiber (%)	0.12	<0.05	0.23
Ash (%)	<0.05	<0.05	<0.05

**Table 2:** Moisture content (%) of non-germinated (day 0) and germinated (day 2–6) peas.

	Day			
Variety	0	2	4	6
Moisture (%) *				
Agassiz	9.5 ± 0.1 ^hi^	10.1 ± 0.1 ^g^	12.3± 0.2 ^e^	14.0 ± 0.1 ^d^
Durwood	9.6 ± 0.1 ^ghi^	9.1 ± 0.0 ^gh^	13.7 ± 0.2 ^d^	19.1 ± 0.1 ^a^
Greenwood	9.6 ± 0.1 ^ghi^	10.1 ± 0.1 ^g^	13.4 ± 0.1 ^d^	16.5 ± 0.3 ^b^
Passion	8.2 ± 0.0 ^j^	10.9 ± 0.1 ^f^	15.6 ± 0.1 ^c^	15.4 ± 0.1 ^c^
Treasure	9.1 ± 0.1 ^i^	10.1 ± 0.1 ^gh^	12.6 ± 0.1 ^e^	18.7 ± 0.6 ^a^

* Data points represent the mean ± standard deviation of two independent experiments. Different letters indicate statistically significant differences (*p* ≤ 0.05) between samples.

**Table 3:** Proximate content (%) of non-germinated (day 0) and germinated (day 2–6) peas.

Variety	Day			
	0	2	4	6
Ash (%) *				
Agassiz	3.0 ± 0.01 ^a^	2.8 ± 0.02 ^b^	2.4 ± 0.01 ^c^	2.5 ± 0.04 ^c^
Durwood	2.3 ± 0.02 ^d^	2.1 ± 0.00 ^fg^	2.0 ± 0.01 ^hi^	1.8 ± 0.04 ^ij^
Greenwood	2.2 ± 0.01 ^def^	2.1 ± 0.04 ^gh^	2.0 ± 0.02 ^hi^	1.9 ± 0.01 ^hij^
Passion	2.2 ± 0.02 ^def^	2.0 ± 0.08 ^h^	1.8 ± 0.01 ^j^	1.9 ± 0.01 ^hij^
Treasure	2.3 ± 0.06 ^d^	2.2 ± 0.01 ^efg^	2.1 ± 0.01 ^gh^	1.9 ± 0.02 ^ij^
Protein (%) *				
Agassiz	22.9 ± 0.1 ^f^	22.4 ± 1.4 ^f^	24.7 ± 1.8 ^ef^	26.1 ± 0.6 ^cde^
Durwood	26.0 ± 1.8 ^de^	28.7 ± 0.4 ^abcd^	28.7 ± 0.2 ^abcd^	31.5 ± 0.1 ^a^
Greenwood	24.9 ± 1.5 ^def^	26.1 ± 0.7 ^de^	26.8 ± 0.2 ^cde^	27.2 ± 1.3 ^bcde^
Passion	27.8 ± 0.6 ^abcde^	27.9 ± 0.5 ^abcde^	30.9 ± 0.5 ^ab^	30.1 ± 1.9 ^abc^
Treasure	25.1 ± 0.1 ^de^	24.3 ± 0.9 ^cde^	27.4 ± 0.3 ^bcde^	28.5 ± 0.7 ^abcde^
Fat (%) *				
Agassiz	1.9 ± 0.0 ^bc^	1.7 ± 0.2 ^cd^	2.1 ± 0.1 ^abc^	2.1 ± 0.2 ^abc^
Durwood	1.4 ± 0.13 ^cdef^	1.3 ± 0.2 ^def^	1.5 ± 0.1 ^cdef^	1.0 ± 0.1 ^f^
Greenwood	2.4 ± 0.2 ^ab^	2.3 ± 0.2 ^ab^	2.4 ± 0.1 ^ab^	2.6 ± 0.1 ^a^
Passion	1.1 ± 0.2 ^ef^	0.9 ± 0.1 ^f^	1.0 ± 0.1 ^f^	1.4 ± 0.3 ^cdef^
Treasure	1.3 ± 0.2 ^cdef^	1.4 ± 0.16 ^cdef^	1.6 ± 0.1 ^cde^	1.0 ± 0.0 ^f^
Total Starch (%) *				
Agassiz	52.3 ± 2.7 ^abcd^	54.6 ± 0.5 ^abc^	55.4 ± 1.4 ^ab^	59.5 ± 0.2 ^a^
Durwood	45.7 ± 1.7 ^def^	46.1 ± 3.6 ^cdef^	46.7 ± 0.8 ^cdef^	43.6 ± 2.6 ^ef^
Greenwood	48.3 ± 0.2 ^bcdef^	47.4 ± 1.3 ^cdef^	48.2 ± 0.5 ^bcdef^	51.7 ± 2.9 ^abcde^
Passion	50.9 ± 0.1 ^bcde^	51.4 ± 3.3 ^abcde^	49.6 ± 0.1 ^bcde^	40.1 ± 4.2 ^f^
Treasure	51.5 ± 1.4 ^abcde^	48.2 ± 0.1 ^bcdef^	49.2 ± 1.5 ^bcde^	50.5 ± 3.5 ^bcde^
Total Dietary Fiber (%) *				
Agassiz	19.9 ± 0.3 ^a^	17.6 ± 1.5 ^ab^	17.5 ± 1.2 ^bc^	14.6 ± 0.2 ^def^
Durwood	16.6 ± 0.3 ^bcd^	15.9 ± 0.4 ^bcdef^	15.3 ± 0.4 ^cdef^	14.2 ± 0.5 ^ef^
Greenwood	17.4 ± 0.5 ^bc^	16.5 ± 0.7 ^bcd^	16.7 ± 0.6 ^bcd^	13.8 ± 0.2 ^f^
Passion	17.5 ± 0.2 ^bc^	16.4 ± 0.2 ^bcde^	15.9 ± 0.2 ^bcdef^	15.0 ± 0.1 ^def^
Treasure	17.8 ± 0.3 ^ab^	15.9 ± 0.2 ^bcdef^	15.0 ± 0.2 ^def^	13.9 ± 0.6 ^f^

* All the data were calculated on a dry weight basis. Data points represent the mean ± standard deviation of two independent experiments. Different letters indicate statistically significant differences (*p* ≤ 0.05) between samples.

**Table 4:** B vitamin concentration (µg/100 g) of non-germinated (day 0) and germinated (day 2–6) peas.

Variety	Day			
	0	2	4	6
	Folate (µg/100 g) *			
Agassiz	352 ± 37 ^bc^	108 ± 5 ^ghi^	140 ± 22 ^fgh^	24 ± 16 ^i^
Durwood	518 ± 26 ^a^	252 ± 13 ^cde^	162 ± 49 ^efgh^	136 ± 34 ^fghi^
Greenwood	458 ± 12 ^a^	231 ± 26 ^de^	177 ± 1 ^efg^	70 ± 20 ^hi^
Passion	445 ± 37 ^ab^	202 ± 4 ^efg^	217 ± 44 ^ef^	96 ± 48 ^ghi^
Treasure	440 ± 4 ^ab^	323 ± 8 ^cd^	460 ± 14 ^a^	261 ± 7 ^cde^
	Niacin (µg/100 g)			
Agassiz	538 ± 89 ^bc^	671 ± 3 ^bc^	683 ± 122 ^bc^	609 ± 91 ^bc^
Durwood	699 ± 260 ^bc^	737 ± 162 ^bc^	535 ± 152 ^bc^	950 ± 37 ^b^
Greenwood	456 ± 27 ^c^	654 ± 140 ^bc^	583 ± 53 ^bc^	654 ± 46 ^bc^
Passion	580 ± 42 ^bc^	545 ± 67 ^bc^	799 ± 30 ^bc^	856 ± 199 ^b^
Treasure	714 ± 8 ^bc^	784 ± 45 ^bc^	922 ± 22 ^b^	1434 ± 28 ^a^
	Pyridoxine (µg/100 g)			
Agassiz	1467 ± 44 ^ab^	187 ± 6 ^ef^	203 ± 41 ^ef^	132 ± 3 ^f^
Durwood	1561 ± 88 ^a^	349 ± 158 ^de^	374 ± 71 ^de^	516 ± 25 ^d^
Greenwood	1151 ± 34 ^c^	258 ± 5 ^ef^	288 ± 24 ^ef^	377 ± 8 ^de^
Passion	1298 ± 51 ^bc^	353 ± 10 ^de^	271 ± 56 ^ef^	174 ± 20 ^ef^
Treasure	1402 ± 45 ^ab^	324 ± 27 ^def^	362 ± 19 ^de^	330 ± 1.0 ^def^
	Riboflavin (µg/100 g)			
Agassiz	156 ± 35 ^abc^	37 ± 2 ^gh^	71 ± 20 ^defgh^	54 ± 19 ^efgh^
Durwood	109 ± 50 ^cdefg^	43 ± 19 ^gh^	70 ± 15 ^defgh^	194 ± 15 ^ab^
Greenwood	119 ± 0 ^cde^	47 ± 6 ^fgh^	76 ± 1 ^defgh^	218 ± 2 ^a^
Passion	122 ± 2 ^cde^	41 ± 8 ^gh^	83 ± 9 ^defgh^	115 ± 28 ^cdef^
Treasure	77 ± 4 ^defgh^	30 ± 6 ^h^	54 ± 5 ^efgh^	142 ± 5 ^bcd^
	Thiamine (µg/100 g)			
Agassiz	209 ± 33 ^c^	251 ± 2 ^bc^	287 ± 44 ^bc^	259 ± 20 ^bc^
Durwood	273 ± 61 ^bc^	278 ± 32 ^bc^	223 ± 49 ^bc^	343 ± 23 ^ab^
Greenwood	207 ± 17 ^c^	249 ± 55 ^bc^	247 ± 7 ^bc^	270 ± 18 ^bc^
Passion	279 ± 15 ^bc^	217 ± 17 ^bc^	292 ± 17 ^bc^	247 ± 71 ^bc^
Treasure	241 ± 10 ^bc^	266 ± 20 ^bc^	310 ± 2 ^abc^	432 ± 2 ^a^

* All vitamin concentrations were calculated on a dry weight basis. Data points represent the mean ± standard deviation of two independent experiments analyzed in duplicate. Different letters indicate statistically significant differences (*p* ≤ 0.05) between samples.

**Table 5:** Effects of the variety, day, and interaction between variables on the in vitro protein digestibility (%) and protein-corrected amino acid score (PDCAAS) of non-germinated and germinated peas.

Measurement	*p* Value		
	Variety	Day	Variety × Day
In vitro protein digestibility (%)	<0.05	<0.05	0.1
IV-PDCAAS (score 0–1)	<0.05	<0.05	<0.05

## Data Availability

The Dissertation of Abdollahikhamene is available through the Public Research Access Institutional Repository and Information (Open Prairie) system at South Dakota State University. Abdollahikhamene, Hojjat. Effect of Six-Day Germination on Chemical Composition, Functional, and Nutritional Properties of Pea Varieties. *Electron. Theses Diss.* **2025**, 1528. https://openprairie.sdstate.edu/etd2/1528 (accessed on 1 June 2025) [108].

## Abbreviations

The following abbreviations are used in this manuscript:

AGA	Agassiz
DW	Durwood
GW	Greenwood
PAS	Passion
TRS	Treasure
IVPD	In vitro protein digestibility
IV-PDCAAS	In vitro protein digestibility corrected amino acid score
DPPH	2,2-Diphenyl-1-picrylhydrazyl
PCA	Principal Component Analysis
TDF	Total dietary fiber
IDF	Insoluble dietary fiber
SDF	Soluble dietary fiber
